# Five-Year Retrospective Study of Uterine STUMP and Leiomyosarcoma

**DOI:** 10.3390/clinpract12060094

**Published:** 2022-11-10

**Authors:** Madalina Bosoteanu, Mariana Deacu, Raluca Ioana Voda, Cristian Ionut Orasanu, Mariana Aschie, Sabina Elena Vlad, Roxana Cleopatra Penciu, Sergiu Ioachim Chirila

**Affiliations:** 1 Clinical Service of Pathology, “Sf. Apostol Andrei” Emergency County Hospital, 900591 Constanta, Romania; 2 Department of Pathology, Faculty of Medicine, “Ovidius” University of Constanta, 900527 Constanta, Romania; 3 Center for Research and Development of the Morphological and Genetic Studies of Malignant Pathology-CEDMOG, “Ovidius” University of Constanta, 900591 Constanta, Romania; 4 Academy of Medical Sciences of Romania, 030167 Bucharest, Romania; 5 Department of Obstetrics and Gynecology, Faculty of Medicine, “Ovidius” University of Constanta, 900527 Constanta, Romania; 6 Medical Informatics and Biostatistics, Faculty of Medicine, Ovidius University, 900527 Constanta, Romania

**Keywords:** uterus, STUMP, leiomyosarcoma, statistics, p53, CD8

## Abstract

Taking into account the unpredictable evolution of uterine STUMP and leiomyosarcomas, there are no clearly established therapeutic protocols to date, the only certified treatment being total hysterectomy. We performed a 5-year retrospective study including cases of malignant tumors and those with uncertain malignant potential originating in the smooth muscle tissue of the uterine body. The clinical data, pathological aspects, and the immunohistochemical results were statistically analyzed using SPSS Statistics Version 26. The main associations of the p53 gene were identified with age, atypia, and the occurrence of metastases. The average number of CD8+ T cells correlated with the hormonal status of the patients, the presence of diabetes, and alteration of thyroid function, but also with the severity of the atypia. The therapeutic method was represented by total hysterectomy, and 30% of the patients with leiomyosarcoma also benefited from adjuvant chemotherapy. The average period until death was 25.66 months. The present study showed that the mutant expression of p53 could have a role in assessing the clinical evolution of patients, given the association with exitus and metastasis. In addition, the average number of CD8+ T cells corresponded to severe atypia, indicating the possibility of applying targeted immunotherapies in these cases.

## 1. Introduction

According to WHO 2020, mesenchymal tumors developed at the level of the uterine body are the second-most frequent tumor category, of which, the most common are the tumors originating at the level of smooth muscle fibers [1]. Depending on mitotic activity, proliferation capacity, cytological atypia, and tumor necrosis or coagulation, they are classified into leiomyomas, leiomyosarcomas, or smooth muscle tumors with uncertain malignant potential (STUMP), respectively [2].

STUMP is a rare pathology which affects approximately 0.01% of patients treated by myomectomy or hysterectomy [3]. Current theories consider this lesional category to be a transitional phase from a leiomyoma to a leiomyosarcoma or to be an undiagnosed low-grade leiomyosarcoma [4]. This pathology is diagnosed more frequently in women during the fertile period, with an average patient age of 44 years [5]. Despite the fact that they are considered to carry a low malignant potential, these tumors have the ability to recur and metastasize. The recurrence rates reported in the literature are between 7% and 27%, with a total recurrence rate of 11% [5,6,7].

Uterine leiomyosarcomas are rare mesenchymal tumors, representing 1–3% of all malignant uterine tumors and approximately 30% of all sarcomas developed in this organ [8]. The prognosis of these tumors depends on FIGO staging. Thus, the 5-year disease-free survival rates for stages I and II are 75.8% and 60.1%, respectively; for stages III and IV, rates are 44.9% and 28.7%, respectively [9]. The treatment of choice is hysterectomy, which can be associated with oncological treatment based on chemotherapy, radiotherapy, or targeted therapies [8].

The aim of the current study is to report the demographic characteristics, the p53 gene mutation, the presence, and the intensity of the inflammatory tumor infiltrate evaluated by the presence of CD8+ T lymphocytes in the cases diagnosed with uterine leiomyosarcoma and STUMP over a period of 5 years in the Dobrogea region.

## 2. Materials and Methods

We performed a retrospective study over a 5-year period that included cases of malignant tumors and those with uncertain malignant potential originating in the smooth muscle tissue of the uterine body. These cases were diagnosed in the obstetrics and gynecology departments of the County Emergency Clinical Hospital of Constanţa.

The information related to clinical data, paraclinical data, treatment, and evolution was extracted from the patient clinical observation forms and the digital medical records of the hospital.

After performing the surgical interventions, the hysterectomy pieces were sent for evaluation in the Clinical Pathological Anatomy Service of the same hospital. Firstly, they were described grossly, taking into account the type of surgery, the maximum diameter of the lesions, and the presence of hemorrhagic and necrotic areas. The fragments were processed by embedding them in paraffin and staining the slides with hematoxylin-eosin. The final diagnosis was established according to the WHO criteria corresponding to the year in which the patients were diagnosed. The cases of interest were re-evaluated by two different doctors, taking into account the criteria of the latest WHO classification.

The immunohistochemical evaluation was performed at the Center for research and development of the morphological and genetic studies of malignant pathology (CEDMOG) using p53 (SP5 clone, dilution 1:50, HIER-DAB method) and CD8 (SP16 clone, dilution 1:50, HIER-DAB method) from Master Diagnostica (Sevilla, ES). The tests were performed on 4-micron-thick paraffin sections according to the manufacturer’s protocols.

The quantification of p53 was carried out as follows: mutant-type immunoexpression with its two variants; overexpression when the reaction is diffuse and intensely positive in at least 80% of the nuclei or null, in which case the immunoreaction is absent; wild-type immunoexpression, considered as normal.

The evaluation of CD8 was carried out qualitatively as well as quantitatively. The quality of the expression was assessed as positive or negative, and the amount of CD8+ T lymphocytes was evaluated by the average number of CD8+ T cells per 1 mm2. To obtain this value, we counted the immunopositive cells from 10 HPF fields (40×) in the hot spot.

Statistical data analysis was performed in SPSS Statistics Version 26 (IBM Corporation, Almonk, NY, USA). Central tendency and variability indicators were used. Univariate data analysis was performed using chi-square test and Fisher’s exact test for categorical data and Mann–Whitney U Test and Kruskal–Wallis H Test for continuous variables, as appropriate. To appreciate the association of data, we used the Pearson correlation coefficient. Survival estimates were made for a period of 5 years and were calculated using the Kaplan–Meier estimator.

All patients signed the informed consent at the time of hospitalization. Furthermore, this study was approved by the Ethics Committee of the hospital.

## 3. Results

In the mentioned period, we identified 829 cases of mesenchymal tumors of the uterine body, of which three cases of STUMP and nine cases of leiomyosarcoma were eligible for our study.

Following the statistical analysis, we observed that the average age of the cases selected for this study was 52.17 years—more precisely, 45 years for STUMP and 54.56 years for leiomyosarcoma. Most of the patients were in the menopausal period, these cases being encountered in the case of the diagnosis of leiomyosarcoma, unlike those diagnosed with STUMP, which were perimenopausal. A statistically significant association was observed between the menopausal period and the diagnosis of leiomyosarcoma, but also between premenopause and the diagnosis of STUMP (*p* = 0.014).

Also, most nulliparous patients were identified among those diagnosed with malignant tumors (55.5%). None of the patients had neither previous hormonal and/or chemotherapeutic treatments, nor significant family history and/or personal pathological history in the gynecological sphere. The most frequent personal pathological antecedents outside the gynecological sphere were dyslipidemia (58.3%), arterial hypertension (50%), diabetes (16.7%), and thyroid function impairment (16.6%). None of the patients has a history of oncological treatment, chemotherapy, or radiotherapy.

The main reasons for hospitalization in both pathologies were metrorrhagia and pelvic pain. Regarding the association of symptoms with laboratory tests, we observed a statistically significant association between the presence of metrorrhagia and patients with dyslipidemia (*p* = 0.014) and between the presence of pelvic pain and those without the aforementioned comorbidity (*p* = 0.040).

The average diameter identified was 5.33 cm in STUMP (3–7 cm) and 11.5 cm in leiomyosarcoma (5–28 cm). The evaluation in the routine staining described, in the majority of cases, the presence of necrosis (88.89%) and the absence of areas of hemorrhage (77.78%). Tumor necrosis was more frequent in patients with arterial hypertension (*p* = 0.049).

**Figure 1:**
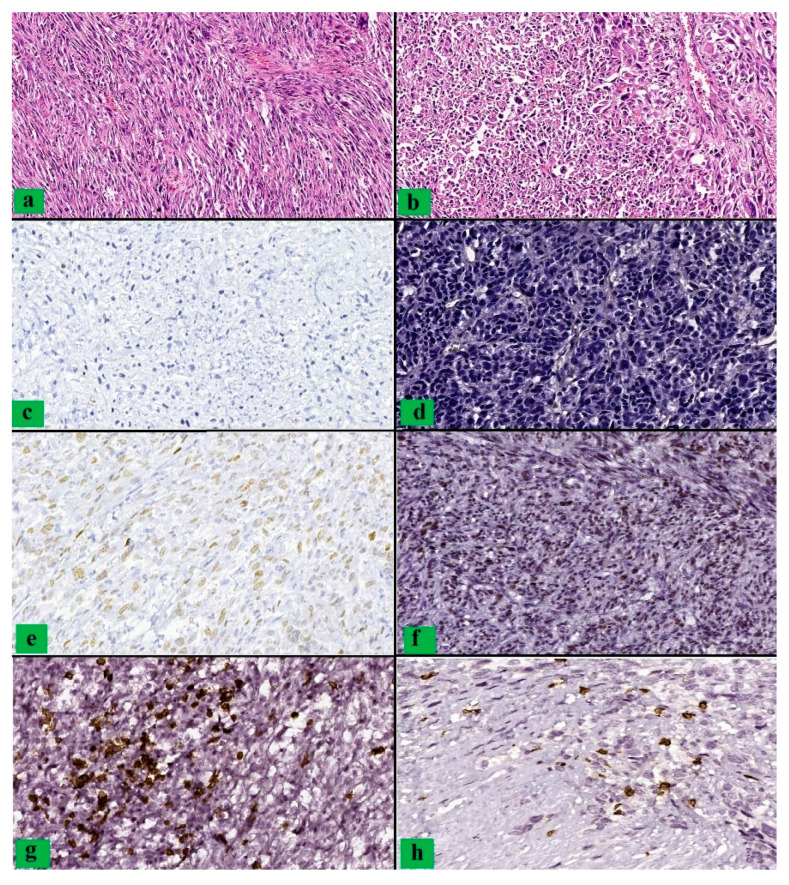
Leiomyosarcoma: (**a**,**b**) microscopic aspects showing a proliferation of malignant smooth muscle fibers with increased density and the presence of atypical mitoses (HE, ob 200×); (**c**,**d**) p53 null-type (ob 200×); (**e**,**f**) p53 overexpressed (ob 200×); (**g**,**h**) CD8 positive T cells (ob 200×).

**Figure 2:**
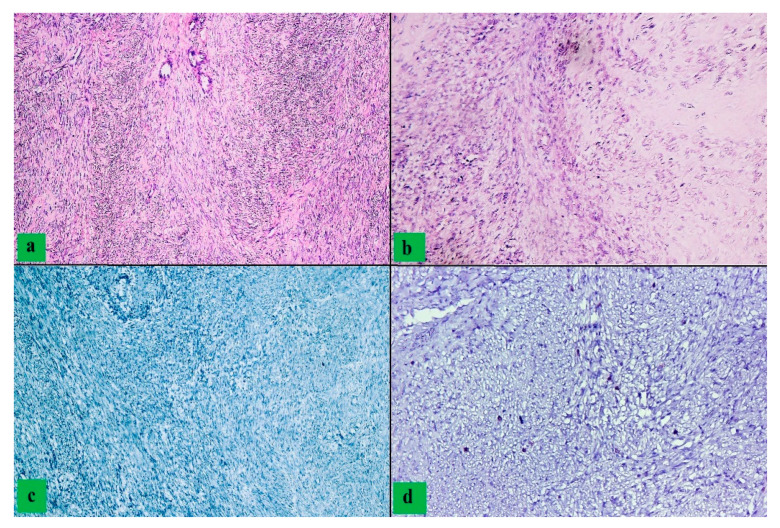
STUMP: (**a**,**b**) proliferation of smooth muscle fibers with increased density and moderate nuclear atypia (HE, ob 200×); (**c**) absence of p53 immunohistochemical expression (ob 200×); (**d**) CD8 positive T cells (ob 200×).

Severe cytoarchitectural atypia were identified only in the case of leiomyosarcomas, insignificant abnormalities were observed only in patients diagnosed with STUMP, and moderate atypia were predominantly identified in cases with malignant tumors (Figure 1 and Figure 2). We observed a statistically significant association between the advanced age and the presence of severe atypia (*p* = 0.019).

**Figure 3:**
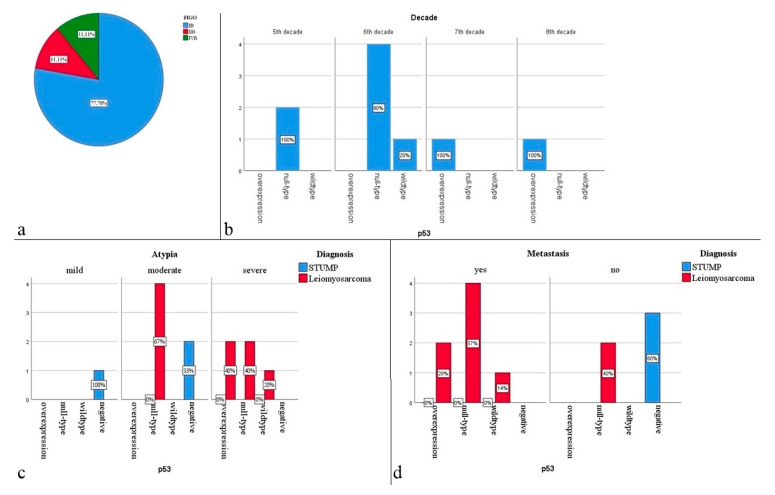
(**a**) FIGO staging distribution; (**b**) the variation of p53 expression according to the decade; (**c**) the variation of p53 expression depending on the cytological atypia and diagnosis; (**d**) the variation of p53 expression according to metastasis and diagnosis.

Lymphovascular invasion was found in 30% of the studied cases diagnosed with leiomyosarcoma, and perineural invasion was absent in all cases. Regarding the staging of the malignant tumors included in the study, the most frequently encountered stage was FIGO IB (77.78%), followed by stages IIB and IVB (11.11% each) (Figure 3a). Of these cases, 77.78% presented metastases.

Concerning the immunohistochemical evaluation, we observed a statistically significant, inversely proportional association between age and the type of p53 immunoexpression (*p* = 0.034). Expressions of the mutant type were found at advanced ages. The overexpression of p53 was observed mainly in the 7th and 8th decades, null expression in the 6th decade, and negative expression in the 5th decade (*p* = 0.016) (Figure 3b). Moreover, there was a statistically significant difference between the percentage of nuclei reactive to p53 and the decade of age, observing a gradually increasing relationship between the two (*p* = 0.039). We identified a statistically significant association between the severity of the atypia and immunoexpression of the p53 marker (*p* = 0.039), severe atypia being more frequent in cases with overexpressed p53 and in the wildtype subgroup (Figure 3c). We identified an association between the occurrence of metastases and the type of p53 immunoexpression so that distant metastasis was observed in all cases with overexpression of the p53 gene and in 66.6% of cases with null mutant expression (*p* = 0.026) (Figure 3d).

**Figure 4:**
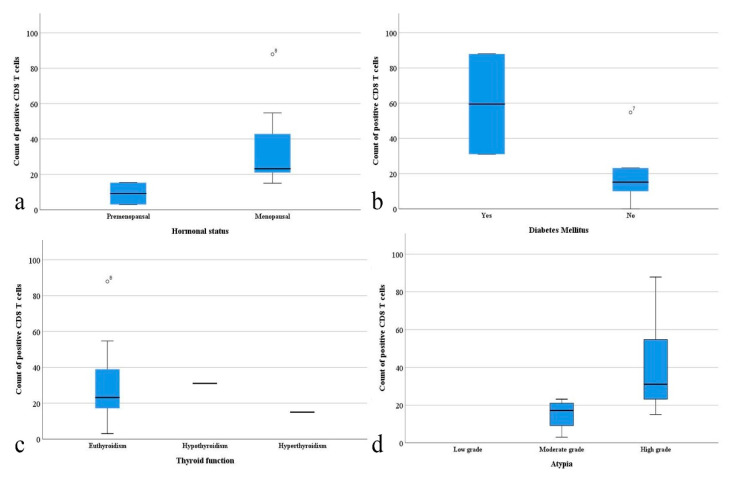
Correlations of the number of CD8 positive T cells with: (**a**) hormonal status; (**b**) diabetes mellitus; (**c**) thyroid function; (**d**) atypia.

Following the statistical evaluation of the CD8 immunohistochemical marker, a higher average number of CD8+ T cells was observed in menopausal patients (*p* = 0.007). The same type of association was observed in the case of patients with diabetes (*p* = 0.018) but also in those with altered thyroid function (*p* = 0.04). A statistically significant association was identified between the severity of the atypia and the positivity of the CD8 marker (*p* = 0.024). The average number of CD8+ T lymphocytes was higher in cases with severe atypia, followed by moderate ones and, eventually, by cases with reduced atypia (*p* = 0.043) (Figure 4).

The first-line treatment in both pathologies was surgical: laparotomic total hysterectomy. Adjuvant chemotherapeutic treatment was performed in 30% of leiomyosarcoma cases, and a correlation with the presence of tumor necrosis areas was observed (*p* = 0.049).

**Figure 5:**
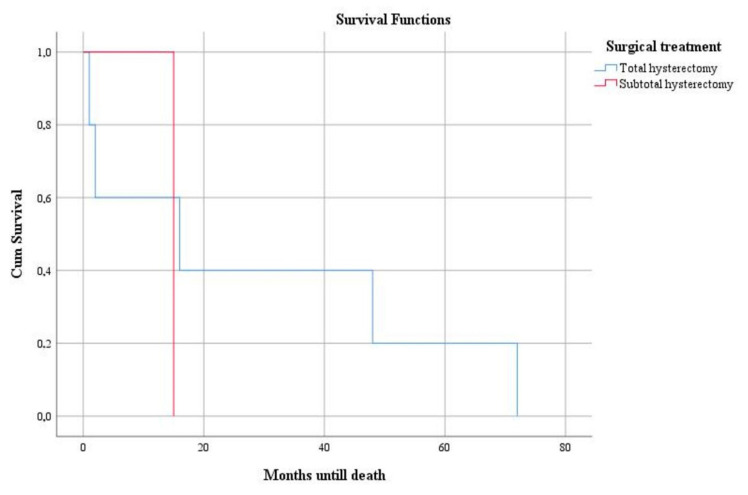
Kaplan–Meier survival graphic that shows a lower survival rate in case of subtotal hysterectomy.

**Figure 6:**
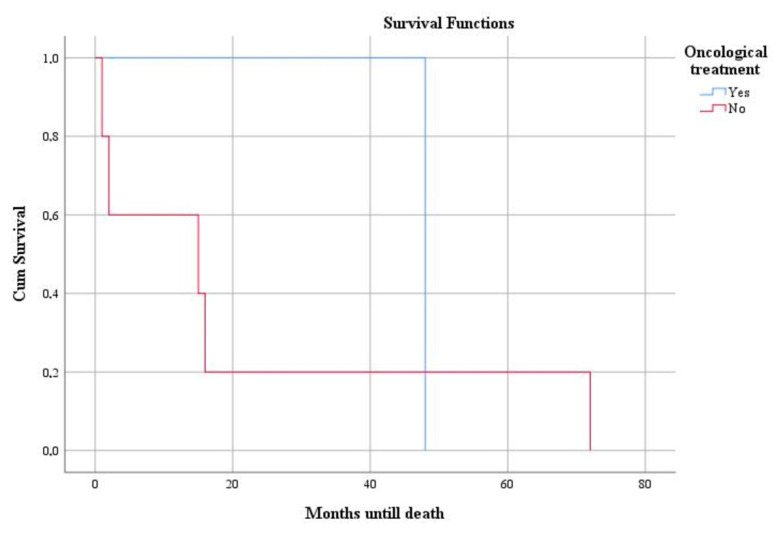
Kaplan–Meier survival graphic that shows a lower survival rate in cases without oncological treatment.

Deaths were recorded in patients diagnosed with leiomyosarcoma after an average period of 25.66 months from the time of diagnosis. We identified a statistically significant association between mutant expression of the p53 gene and higher mortality rate (*p* = 0.008). Survival was elevated in patients treated by total hysterectomy (Figure 5) and adjuvant chemotherapy (Figure 6) but was without statistical significance (*p* = 0.583 and *p* = 0.646, respectively).

## 4. Discussion

The spectrum of uterine smooth muscle tumors varies from the benign variants represented by leiomyoma to the malignant equivalent, leiomyosarcoma [10]. A similar tumor that cannot be certainly diagnosed as benign or malignant is called STUMP. The evolution of this category is what confers the final biological potential [11].

STUMP tumors are characterized by clinical and histological heterogeneity [12]. They have a frequency of approximately 0.01%, their prevalence being difficult to assess due to their rarity and variable/subjective diagnostic criteria [12,13]. The incidence of uterine leiomyosarcoma is between 0.35–0.8/100,000, with a prevalence of 3–7/100,000 [14,15,16]. The scarcity of this pathology was also identified in our study, establishing a frequency of 0.36% in STUMP and 1.08% in leiomyosarcoma.

The clinical manifestations of STUMP are nonspecific, being frequently similar to those of leiomyomas—namely metrorrhagia, pelvic mass, and pelvic pain—but also secondary symptoms of compression phenomena or anemia [12]. Regarding leiomyosarcomas, the clinical manifestations at the time of diagnosis are represented by metrorrhagia (56%), palpable pelvic mass (54%), abdominal pain (22%), but also abnormal vaginal secretions, pollakiuria, constipation, and abdominal distension [17,18]. Other less common symptoms are hemoperitoneum or symptoms generated by extrauterine extension or distant metastases [18]. In our evaluation, the main clinical manifestations that led to medical referral in both of the studied pathologies were represented by metrorrhagia and abdominal pain.

One of the most common subtypes of malignant mesenchymal tumors is represented by leiomyosarcoma. It includes 10–20% of all newly diagnosed soft tissue sarcomas. The most common locations are the abdomen, retroperitoneum, uterus, and large blood vessels [19].

The diagnostic criteria of uterine leiomyosarcoma are represented by nuclear atypia, the number of mitoses, and the presence of tumor necrosis. Interpretation of the type of necrosis can be difficult, especially in the case of small foci. Thus, more emphasis should be placed on nuclear atypia and the number of mitoses [20]. In our case, a statistically significant association was observed between the severity of atypia and the microscopic diagnosis (*p* = 0.033). The number of mitoses must be over 15/10 HPF to be diagnostic for this tumor category [21]. In our study, the average number of mitoses was 15.56/10 HPF.

According to the literature, over 75% of leiomyosarcomas at the time of diagnosis have a maximum diameter greater than 5 cm [14]. In our case, the average diameter at the time of diagnosis was 11.5 cm, with a minimum value of 5.5 cm reported in a single case, the rest having a diameter over 7 cm with a maximum value of 28 cm.

Lymphovascular invasion is a rare phenomenon in the case of uterine leiomyosarcomas, being identified in 10–20% of cases [22]. The same aspect was observed in our study, with lymphovascular invasion being present in 30% of cases.

Approximately 60% of cases are diagnosed when the tumor is limited to the uterus [23]. The study conducted by Tan TS et al. revealed a percentage of 71% of cases diagnosed in stage I of the disease, 19% in the last stage, and 7% in stage II [24]. Among the cases studied by us, the first stage of the disease was identified in 77.8% of patients, followed by 11.1% for stages II and IV.

Mitotic activity is closely related to the occurrence of recurrences in a directly proportional manner: the more identified mitoses, the higher the probability of recurrence [25].

The average period of occurrence of recurrences in the case of STUMP is 51 months, ranging from 15 months to 9 years [25]. In our study, after a period of at least 4 years of cohort follow-up, no recurrences or metastases were objectified in any case.

According to the study carried out by Nassif EF et al., the only molecular alteration associated with disease-free survival was that of the TP53 gene, the lowest survival values being observed in cases with deleted or mutated TP53 [26]. This idea is also supported by the study by Choi J et al., in which they observed that TP53 mutations tend to reduce survival rates [27]. Our study is in accordance with these data, demonstrating that there is an association between mutant p53 expression and patient survival (*p* = 0.008). We observed that those with overexpressed p53 survived 32 months and those with null expression 22.5 months.

Regarding p53 reactivity in STUMP, in the literature, the percentage of cases with positive expression varies between 0–42.3% (Table 1) [5,25,28,29,30,31].

Additionally, the loss of p53 function determines the alteration of the inhibition pathways of metastasis. It is known that transcriptionally deficient TP53 mutants have additional roles that promote metastasis. P53 directly influences the transcription of genes responsible for the appearance of secondary determinations by binding the promoters of a variety of genes with a role in regulating cell motility and adhesion. The loss of p53 seems to contribute to the weakening of intercellular junctions and the destruction of the integrity of epithelia, thus participating in the dissemination of cells from solid tumors [32]. According to the literature, the percentage of cases with abnormal expression of p53 can reach up to 97% (Table 1) [33,34,35,36,37,38]. In the case of our study, we observed an association between the type of p53 immunoexpression and the occurrence of secondary determinations. Distant metastasis was observed in all cases with overexpression of the p53 gene and in 66.6% of cases with null mutant expression (*p* = 0.026).

The presence of specific killer lymphocytes in the tumor microclimate was defined as tumoral lymphocytic infiltrate [39]. Studies have shown that CD8-positive cytotoxic T lymphocytes are essential for controlling tumor development, their presence being associated with the favorable evolution of several malignant neoplasms. These cells inhibit tumor growth through the ability to kill tumor cells through perforins, granzymes, and other cytokines. For example, in endometrial carcinomas, the presence of these lymphocytes in the lymphocytic tumor infiltrate is associated with a better prognosis [40]. Data from the literature related to the involvement of CD8 in the evolution of these tumors are few, and we want to enrich the literature by finding that the average number of CD8/mm^2^ was statistically significantly associated with severe atypia. Comparing the two tumor types, in the case of carcinomas, the maximum activity of CD8 is related to tumor growth, while in our case, the number of CD8 was closely related to severe atypia without these having determined tumor growth (*p* = 0.269) [40].

Related to the therapeutic approach of STUMP, both the laparoscopic method and laparotomy can be used for myomectomy or hysterectomy. Morcellation could be used in both situations, but it can lead to the appearance of metastases [25]. The cases diagnosed with STUMP in the present study were treated by total hysterectomy, keeping the tumoral lesion intact.

Total hysterectomy is the treatment of choice for uterine leiomyosarcoma and does not involve morcellation or intraoperative rupture of the tumor [8]. Preservation of the ovaries could be considered in premenopausal patients to preserve hormonal function, especially in stages I and II of the disease [20]. Adjuvant radiotherapy is ineffective, and there is no established therapeutic regimen for adjuvant chemotherapy [41]. The standard treatment for resectable cases is total hysterectomy with bilateral abdominal adnexectomy, and for unresectable, advanced, or recurrent cases, the therapeutic option is chemotherapy [41]. In our study, the initial therapeutic behavior was represented by laparotomic total hysterectomy in most cases, with only two cases being surgically treated by total hysterectomy with bilateral adnexectomy and subtotal hysterectomy, respectively. Later, a third of the cases received chemotherapy treatment.

As a rule, STUMP is characterized by indolent behavior and prolonged survival, although cases with an accelerated clinical evolution due to distant metastases have also been reported [42,43,44]. In the study by Rizzo A et al., survival after the initial diagnosis was on average 101 months [45]. In our study, no deaths or metastases were reported in patients with the same diagnosis.

Considering leiomyosarcomas, survival rates after a period of 5 years vary from 25% to 76%, dropping to 10–15% if distant metastases are present at the time of initial diagnosis [14]. In our analysis, 4 years after the time of diagnosis, the mortality rate registered values of 66.67%, with an average survival of 25.66 months.

The limitation of this study is the small number of cases studied, but the strengths of this study are represented by the rarity of the tumors studied and the absence in the literature of other studies on the same topic. From our point of view, such studies should be performed on larger groups of patients in centers specialized in gynecological pathology.

## 5. Conclusions

After performing statistical analysis, our study supports the data from the literature, bringing forward new information regarding the correlation between the biological behavior, the p53 suppressor gene, and the reaction of CD8-positive T lymphocytes. Thus, the present study showed that the mutant expression of p53 could have a role in assessing clinical patient evolution given its association with exitus and metastasis. In addition, the average number of CD8+ T cells corresponded to severe atypia, indicating the possibility of applying targeted immunotherapies in these cases.

## Figures and Tables

**Table 1:** The main studies analyzing the immunoreaction of the p53 marker in cases of uterine leiomyosarcoma and STUMP. [5,25,28,29,30,31,33,34,35,36,37,38].

Diagnosis	Author (Year)	Number of Cases	Average Age	Average of Maximum Diameter (cm)	p53 Positive Reaction	Myomectomy (%)/Hysterectomy (%)	Recurrences	Menopausal/Premenopausal
**STUMP**	Ning C et al. (2021) [28]	16	45	NS **	0%	37.5%/62.5%	6.3% (STUMP)	25%/75%
	Huo L et al. (2020) [25]	26 */67	42	7	27% (26 cases)	56.7%/43.3%	15% (leiomyosarcoma, STUMP)	8.9%/91.1%
	Zheng YY et al. (2020) [29]	26	42.96	8.2	42.3%	26.9%/73.1%	23% (STUMP)	NS **
	Yordanov AD et al. (2020) [5]	14	45.4	7.5	NE ***	16.7%/85.7%	0%	7.1%/92.9%
	Şahin H et al. (2019) [30]	57	42	6	0%	47.3%/52.7%	14% (leiomyosarcoma, STUMP)	NS **
	Ha HI et al. (2018) [31]	19	41	9.5	NE ***	52.6%/47.4%	10.5% (leiomyosarcoma, STUMP)	NS **
**Leiomyosarcoma**	Baek MH et al. (2018) [33]	42	47	7.8	38%	NS **	54.8%	64.3%/35.7%
	Zhang Q et al. (2018) [34]	38	55.3	10.5	39%	3%/97%	57%	NS **
	Cuppens T et al. (2017) [35]	84	57	9.7	97%	NS **	NS **	NS **
	Makinen N et al. (2016) [36]	52	58.55	1.5–30	66%	NS **	53.8%	NS **
	Zhou Y et al. (2015) [37]	36	NS **	~5	44.1%	0%/100%	50%	NS **
	Zhang Q et al. (2014) [38]	38	55.3	10.5	24%	3%/97%	60%	NS **

* Eligible cases; ** NS—not specified; *** NE—not evaluated.

## Data Availability

Not applicable.
